# The new Italian registry of infantile thrombosis (RITI): A reflection on its journey, challenges and pitfalls

**DOI:** 10.3389/fped.2023.1094246

**Published:** 2023-04-20

**Authors:** Maria Federica Pelizza, Matteo Martinato, Anna Rosati, Margherita Nosadini, Paola Saracco, Paola Giordano, Matteo Luciani, Laura Ilardi, Donatella Lasagni, Angelo Claudio Molinari, Rossana Bagna, Antonella Palmieri, Luca Antonio Ramenghi, Massimo Grassi, Mariella Magarotto, Federica Magnetti, Andrea Francavilla, Giuseppe Indolfi, Agnese Suppiej, Chiara Gentilomo, Roberta Restelli, Antonella Tufano, Daniela Tormene, Jacopo Norberto Pin, Clarissa Tona, Davide Meneghesso, Lidia Rota, Marta Conti, Giovanna Russo, Giulia Lorenzoni, Dario Gregori, Stefano Sartori, Paolo Simioni, Collaborators of the R.I.T.I. (Italian Registry of Infantile Thrombosis), Accorsi Patrizia

**Affiliations:** ^1^ Paediatric Neurology and Neurophysiology Unit, Department of Women's and Children's Health, University Hospital of Padova, Padova, Italy; ^2^ Master in Pediatrics and Pediatric Subspecialties, University of Padova, Padova, Italy; ^3^ Unit of Biostatistics, Epidemiology and Public Health, Department of Cardiac-Thoracic-Vascular Sciences and Public Health, University of Padova, Padova, Italy; ^4^ Department of Statistics, Computer Science, Applications “G. Parenti”, University of Firenze, Firenze, Italy; ^5^ Neuroscience Center of Excellence, Children's Hospital Anna Meyer, University of Firenze, Firenze, Italy; ^6^ Paediatric Haematology Unit, Department of Paediatrics, University Hospital “Città Della Salute e Della Scienza”, Torino, Italy; ^7^ Department of Biomedical Science and Human Oncology, Aldo Moro University of Bari-Giovanni XXIII Hospital, Bari, Italy; ^8^ Department of Paediatric Hemato-Oncology and Cell and Gene Therapy, Bambino Gesù Children's Hospital, Roma, Italy; ^9^ Neonatal Intensive Care Unit, Niguarda Ca’ Granda Hospital, Milano, Italy; ^10^ Paediatric Unit, Children's Hospital Anna Meyer, University of Firenze, Firenze, Italy; ^11^ Regional Reference Center for Hemorrhagic Diseases, IRCCS Giannina Gaslini Children's Hospital, Genova, Italy; ^12^ Neonatal Intensive Care Unit, University Hospital “Città Della Salute e Della Scienza”, Torino, Italy; ^13^ Department of Paediatric Emergency, IRCCS Giannina Gaslini Children's Hospital, Genova, Italy; ^14^ Neonatal Intensive Care Unit, IRCCS Giannina Gaslini Children's Hospital, Genova, Italy; ^15^ Neonatal Intensive Care Unit, Department of Women's and Children's Health, University Hospital of Padova, Padova, Italy; ^16^ NEUROFARBA Department, Children's Hospital Anna Meyer, University of Firenze, Firenze, Italy; ^17^ Section of Pediatrics, Department of Medical Sciences, University of Ferrara, Ferrara, Italy; ^18^ Paediatric Unit, “Dell'Angelo” Hospital, Venezia, Italy; ^19^ Regional Reference Centre for Coagulation Disorders, Department of Clinical Medicine and Surgery, Federico II University Hospital, Napoli, Italy; ^20^ General Internal Medicine and Thrombotic and Haemorrhagic Unit, University Hospital of Padova, Padova, Italy; ^21^ Paediatric Nephrology, Dialysis and Transplant Unit, Department of Women's and Children's Health, University Hospital of Padova, Padova, Italy; ^22^ Cardiovascular Prevention Centre, Humanitas Research Hospital, Milano, Italy; ^23^ Child Neurology Unit, Department of Neuroscience, Bambino Gesù Children's Hospital, Rome, Italy; ^24^ Unit of Pediatric Onco-Haematology, Department of Clinical and Experimental Medicine, University of Catania, Catania, Italy

**Keywords:** thrombosis, stroke, children, pediatric, registry, thromboembolism, neonatal

## Abstract

**Introduction:**

Thrombotic events in neonates and children represent a rare although severe occurrence in view of the associated risk of mortality and sequelae. Quality evidence is limited in this field, and registry studies provide an essential base for research. The aim of this paper is to present the new Italian Registry of Infantile Thrombosis (RITI), set it into the scene of international thrombosis and stroke registries, and provide some insight on the challenges associated with registry management.

**Methods:**

We present the detailed structure and content of the new RITI registry, a brief overview of its main data, and a reflection on its features, pitfalls and the main challenges related to its management.

**Results:**

The RITI, initially started in 2007 and officially re-launched in 2017 after structural modifications, is a non-interventional retrospective and prospective registry study collecting data on neonatal and pediatric patients (0–18 years) who experienced a systemic or cerebral thrombotic event in Italy. The RITI is managed by a multidisciplinary team with expertise in pediatric thrombosis, and participation is open to all Italian physicians, on a voluntary basis. The overall aim of the registry is to acquire new evidence to better characterize the population of children with thrombotic events and improve their management and outcome. 48 Italian pediatric and intensive care units are actively involved in the RITI, including 85 medical doctors from 16 Italian regions. A total of 1,001 neonates and children affected by cerebral or systemic thrombosis have been enrolled.

**Discussion:**

The RITI is one of the largest available European registries of neonatal and pediatric thrombosis. National registries like the RITI represent a model for the study of rare conditions based on multidisciplinary and multicenter collaboration, aimed at overcoming the limitations due to small populations of patients, and creating a network of experts for patient referral and continuous education. Moreover, registry studies have a pivotal role in the research on pediatric thrombosis, due to the limited feasibility of high-quality studies. In our experience, the main critical stages, pitfalls and challenges in registry management include adequate registry designing, diffusion, data completeness and quality control.

## Introduction and background

Thrombosis in children can be distinguished according to the age of the patient (neonatal or pediatric), the site (cerebral or systemic) and the vascular compartment involved (arterial or venous). Overall, pediatric thrombosis is rare, with incidence varying according to patient's age and thrombosis type (Table 1) (1–14); although, some thrombosis types such as hospital-acquired pediatric venous thromboembolism seem to have undergone a certain increase in incidence over the last decades, probably due to the prolonged survival of children with severe or chronic disease, more invasive care, greater availability of imaging techniques, and higher level of suspicion for thrombosis (15).

**Table 1:** Incidence of neonatal and pediatric thrombotic events.

	Systemic thrombosis		Cerebral thrombosis^b^	
	Arterial	Venous	Arterial	Venous
≤28 days of life^a^	5.1/100,000 live births 2.4–6.8/1,000 NICU admissions	5.1/100,000 live births 2.4–6.8/1,000 NICU admissions	1/2,300–5,000 live births	0.6–12/10,000 live births
>28 days of life	0.2–0.3/100,000 person years	0.7–2/100,000 person years	1–2/100,000 person years	0.3–0.6/100,000 person years

NICU, neonatal intensive care unit.

^a^ Neonatal systemic venous and arterial thrombosis includes events occurring in the first 28 days after birth or up to 44 weeks after conception after premature birth (9–11); perinatal ischemic stroke, instead, is defined as a cerebrovascular insult occurring from 20 weeks of fetal life to 28 days postnatally or 44 weeks postconception in preterm neonates (4, 12–14).

^b^ Pediatric stroke is defined as an acute onset of neurological symptoms caused by focal brain infarction or hemorrhage (4, 5). Ischemic stroke includes arterial ischemic stroke (AIS) and venous infarction due to cerebral sinovenous thrombosis (CSVT) or cortical vein thrombosis (4).

Neonatal and pediatric thrombosis is usually secondary to underlying, and often multiple, conditions or triggers (Table 2) (1–3, 8–10, 16–24), although a specific risk factor is not always identified. The risk of mortality and sequelae varies according to patient's age, thrombosis type, comorbidities and other factors, but it is overall a cause of concern.

**Table 2:** Leading risk factors related to neonatal and pediatric thrombosis.

Major risk factors and causes			
Systemic thrombosis	Arterial	≤28 days of life	Arterial catheterization
		>28 days of life	Arterial catheterization
	Venous	≤28 days of life	Intravascular catheter, systemic infections
		>28 days of life	Intravascular catheters, surgery, immobility, cardiac disease, thrombophilia, infections, malignancy, combined oral contraceptive pills
Cerebral thrombosis	Arterial	≤28 days of life	Maternal and neonatal conditions (e.g., intrapartum fever, preeclampsia, oligohydramnios, operative delivery, emergency caesarean section, fetal distress, resuscitation at birth, hypoglycemia, small for gestation age), thromboembolism from placenta
		>28 days of life	Arteriopathy, cardiac disease, infections, metabolic disorders, prothrombotic disorders, systemic disorders (such as systemic lupus erythematosus)
	Venous	≤28 days of life	Acute systemic disease, infections, dehydration, coagulation disorders, gestational or delivery complications
		>28 days of life	Head and neck disorders, dehydration, acute systemic diseases, infections (e.g., otitis media or mastoiditis), chronic systemic diseases (e.g., inflammatory bowel disease, cancer, autoimmune disorders, chronic kidney disease), prothrombotic states

Research on neonatal and pediatric thrombosis is characterized by the lack of quality data due to the rarity of the condition and the limited feasibility of randomized controlled trials in this clinical setting and age. On the other hand, data derived from studies on adults can only partially be transposed to children and neonates due to the obvious differences in these age groups as regards physiology, clinical symptoms, and pharmacokinetics/pharmacodynamics.

In the last decades, national and international registries have been developed to increase knowledge and awareness of childhood thromboembolism, and represent a core essential tool to progress research by allowing the pooling of larger numbers of patients than single-center and multi-center studies would allow. Table 3 summarizes the main features of a selection of national and international registries on pediatric thrombosis (2, 17, 25–33).

**Table 3:** Selected main national and international registries on pediatric thrombosis.

Registry (oldest and most recent publications)	Registries with focus on cerebral and systemic thrombosis		Registries with focus on cerebral thrombosis only				Registries with focus on systemic thrombosis only	
	Italian Registry of Infantile Thrombosis (RITI) (24, 25)	Dutch Pediatric Surveillance Unit (DPSU) registry (2)	International Paediatric Stroke Study (IPSS) (26, 27)	Canadian Pediatric Ischemic Stroke Registry (CPISR) (8, 17)	Swiss Neuro-Paediatric Stroke Registry (SNPSR) (28, 29)	Save ChildS Pro Registry (30)	Children's Hospital-Acquired Thrombosis (CHAT) Consortium Registry (31, 32)	Throm-PED (33)
Country	Italy	The Netherlands	International	Canada	Switzerland	International	U.S.A.	International
Neonatal/Pediatric age	Neonatal and pediatric (0–18 years)	Neonatal and pediatric (0–18 years)	Neonatal and pediatric (0–18 years)	Neonatal and pediatric (0–18 years)	Neonatal and pediatric (0–16 years)	Pediatric (<18 years)	Neonatal and pediatric (0–21 years)	Neonatal and pediatric (0–18 years)
Year of registry inception	2007 (new registry from 2017)—ongoing	1997–1999	2003—ongoing	1992—ongoing	2000—ongoing	2020—ongoing	2014—ongoing	n.a.—ongoing
Year of occurrence of first thrombotic events included	1996	1997	2003	1992	2000	2020	2012	n.a.
Type of thrombosis included	Cerebral (AIS, CSVT) and systemic (arterial, venous, intracardiac)	Cerebral and systemic VTE	Cerebral (AIS, CSVT)	Cerebral (AIS, CSVT)	Cerebral (AIS, CSVT)	AIS with confirmed arterial occlusion, with endovascular treatment attempted or best medical treatment including IV thrombolysis	Hospital-acquired venous thromboembolism (HA-VTE) + non-VTE hospitalized controls	Systemic
Main aims of the registry	To collect data on the characteristics of neonatal and pediatric thrombosis in Italy, their diagnosis, management and outcome, ultimately to identify the major areas of need in this field, improve their knowledge and awareness in Italy; to lay the foundations for future studies on neonatal and pediatric thrombosis	To assess the current incidence, signs and symptoms, diagnostic tests, risk factors, therapy, and complications of pediatric extremity and nonextremity VTE in The Netherlands	To develop multinational clinical trials in pediatric ischemic stroke	To obtain comprehensive prospective epidemiologic data on stroke, including CSVT, in children	To collect data on manifestations, risk profile, neuroimaging, treatment and outcome of pediatric stroke	To generate evidence for the use of MT in childhood stroke under the hypothesis that MT is safe and results in a high rate of good clinical outcomes compared to the best medical treatment including intravenous thrombolysis	To identify risk factors for HA-VTE, to create a HA-VTE risk prediction model	To increase knowledge on epidemiology, risk factors, diagnosis, treatment, outcome of thrombosis, with focus on specific TE events such as neonatal renal vein thrombosis, portal vein thrombosis, pulmonary embolism, catheter-related thrombosis and arterial thrombosis; to investigate antithrombotic agents use, safety and efficacy in “real life”
Patients/Events	Patients	Patients	Patients	Patients	Patients	Patients	Patients	Patients
Study design	Retrospective and prospective (observational)	Prospective	Retrospective and prospective	Prospective	Prospective	Prospective	Retrospective and prospective (case-control)	Prospective (observational)
Voluntary/mandatory participation	Voluntary	Mandatory (surveillance system developed by the Dutch Pediatric Surveillance Unit)	Voluntary	Voluntary	Mandatory	Voluntary	Voluntary	Voluntary
Methods of data collection and web platform used (if applicable)	Web-based interface (Research Electronic Data Capture, REDCap)	Questionnaire or anonymous discharge letter provided to the Dutch Pediatric Surveillance Unit (monthly notification cards sent to all pediatricians in primary and secondary care centers and contact persons in tertiary care centers to register all children meeting the various case definitions)	Standardized IPSS data collection forms	Standardized data collection forms filled in by a research nurse who visited each center	Questionnaire sent to every neuropaediatrician in Switzerland monthly, and to the neonatal units every three months	Web-based interface (Eppdata)	Web-based interface (Research Electronic Data Capture, REDCap)	Web-based interface (Research Electronic Data Capture, REDCap)
Number of patients enrolled^a^	1,001	115	5,492 (1964 neonatal + 3,528 pediatric)	1,129 AIS + 325 CSVT	>800: 436 AIS	n.a.	1,185 HA-VTE + 884 non-VTE controls	400
Number of centers involved^a^	48 from 16 Italian regions	23	96 in 26 countries across 5 continents	16	14	50 in 9 countries	9	n.a.
Clinical scales used	Pediatric National Institute of Health Stroke Scale (pedNIHSS) Pediatric Stroke Outcome Measure (PSOM)	n.a.	Pediatric Stroke Outcome Measure (PSOM)	Descriptive	Pediatric Stroke Outcome Measure (PSOM)	Modified Rankin Scale (mRS)	n.a.	n.a.

AIS, arterial ischemic stroke; CSVT, cerebral sinovenous thrombosis; EVT, endovascular treatment; HA-VTE, hospital-acquired venous thromboembolism; IV, intravenous; MT, mechanical thrombectomy; n.a., not available; TE, thromboembolism; VTE, venous thromboembolism.

^a^ According to the most recent available publications.

The main aim of the present paper is to present the new Italian Registry of Infantile Thrombosis (RITI), one of the largest available registries on thrombosis in neonates and children in Europe, and to reflect on its journey so far, including its main features and pitfalls.

## Overview of the RITI registry

### RITI history

A first version of the RITI was initially created in 2007, collecting data on thrombotic *events*. The RITI was designed and created thanks to the joint effort of a multidisciplinary team based in Padova, Italy, with expertise in thrombosis and coagulation disorders, pediatrics, neonatology, neurology, hematology, oncology, cardiology, nephrology, and intensive care. Beside health care providers, a team of informatics and statisticians has played an essential role in all stages of the registry creation, maintenance and updates. The study received Ethical Committee approval on May 12th, 2008 (Protocol #1653P), and the RITI was soon opened to all physicians on the Italian territory, who were invited to participate and enroll patients on a voluntary basis. Periodic national in-person meetings were initially held to discuss registry-related issues and promote the awareness of neonatal and pediatric thrombosis.

On 30th June 2017, a new version of the RITI was officially launched, prospectively collecting data on *patients* with thrombosis rather than on thrombotic *events*. This change was deemed strongly necessary in order to allow for a more accurate collection of follow-up data and to conform to the main international and national registries (Table 3).

### Aims of the RITI

In view of the different ages involved (neonatal and pediatric), and the inclusion of both cerebral and systemic thrombosis, both in the arterial and venous compartments, the RITI scope is broad, and include:

i) to improve the understanding on neonatal and pediatric thrombosis in Italy, their clinical features, diagnosis, management and outcome, including efficacy and safety of treatments used;
ii) to increase awareness and identify the major areas of need in this field, in order to lay the foundations and guide the scope of future studies on neonatal and pediatric thrombosis;
iii) to create a network of Italian specialists who can serve as a reference point for “spoke hospitals” to improve the care of patients with thrombosis.

### Study design and inclusion criteria

The RITI is a non-interventional retrospective and prospective registry, collecting anonymized data on neonatal and pediatric patients (age 0–18 years) who experienced a systemic or cerebral thrombotic event in the arterial or venous compartment, in Italy [in particular, arterial ischemic stroke (AIS), cerebral sinovenous thrombosis (CSVT); systemic venous, arterial and intracardiac thrombosis]. No additional diagnostic or therapeutic procedures are required after inclusion in the RITI besides those relative to the standards of good clinical practice. Internationally agreed definitions are used for the diagnosis of thrombotic events (cerebral or systemic, venous or arterial), and patient treatment occurs according to available international and local guidelines, also likely partly influenced by the treating physician's experience and assessment of a patient's risk of bleeding.

### Patient enrolment

Participation to the RITI is open to all physicians on the Italian territory, on a voluntary basis. Inclusion in the RITI registry is subject to written informed consent by the patient's family; the consent form can be found online (https://www.trombosiinfantili.info/). Enrolling physicians are requested to insert both data on the acute phase of the thrombotic event and follow-up data.

### Patient data collection and web platform

Data is directly entered into the RITI platform by the treating physicians, after physician's registration in the RITI (https://www.trombosiinfantili.info/) and identity verification. Data collection system is based on two separate web-based platforms to store personal and clinical data separately, in compliance with the General Data Protection Regulation. Site personnel access a secure web-based platform used for patients’ registration and pseudonymization. Access is protected by a personal username and password.

The patient's personal data are entered into the system and a unique alphanumeric code is generated for each patient. The alphanumeric code serves as the subject's identifier on the web-based platform used for the collection of clinical data.

Study data are collected and managed using REDCap, (Research Electronic Data Capture) (34, 35) a tool hosted at the Department of Cardiac-Thoracic-Vascular Sciences and Public Health, University of Padova, a secure web-based software platform designed to support data capture for research studies, providing (1) an intuitive interface for validated data capture, (2) audit trails for tracking data manipulation and export procedures, (3) automated export procedures for seamless data downloads to common statistical packages, and (4) procedures for data integration and interoperability with external sources.

## Registry structure

**Figure 1:**
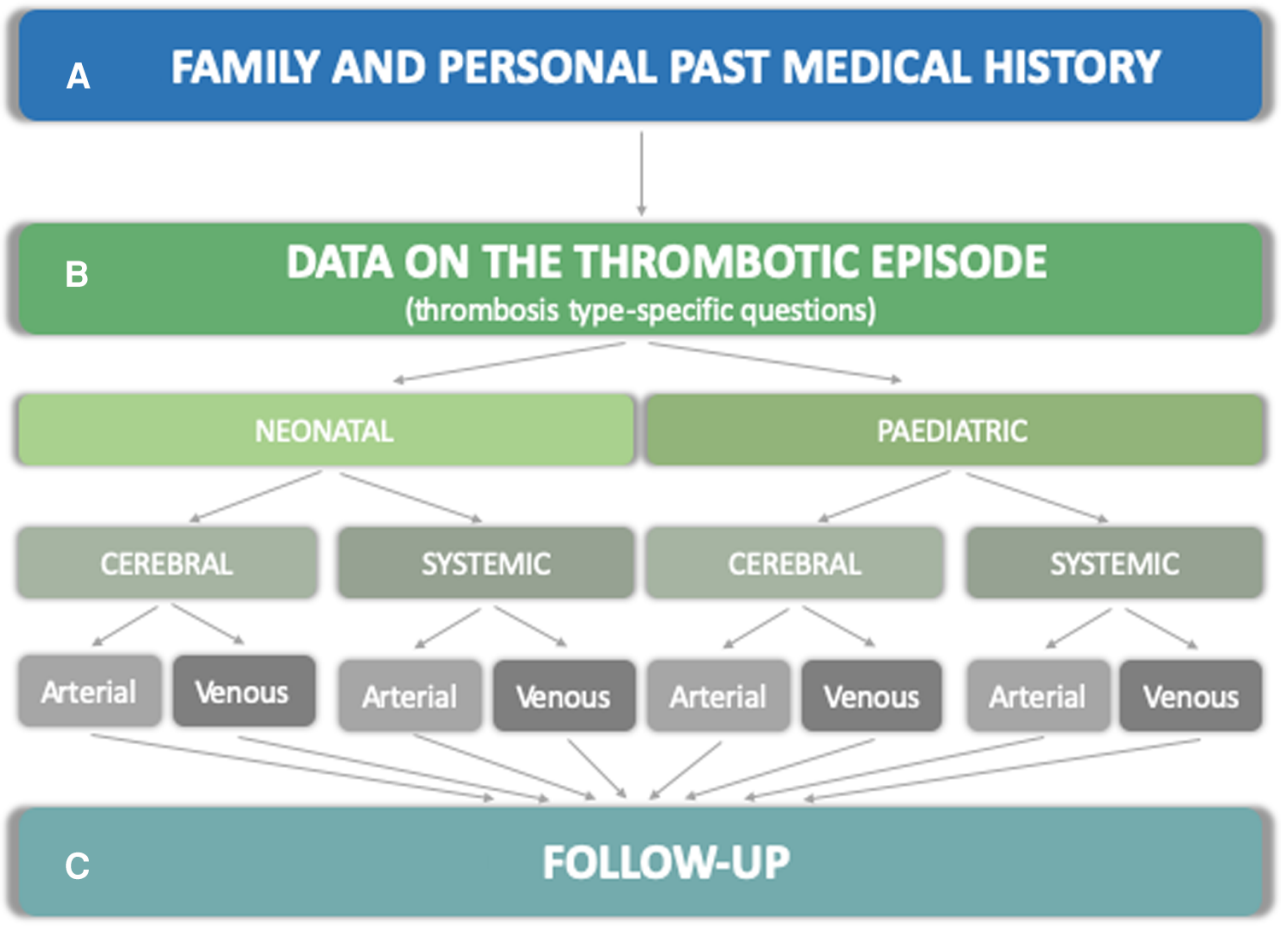
Structure of the data collection form in the RITI registry. The structure of the registry is articulated into sections A (family and past medical history) B (thrombosis-specific questions on risk factors, clinical and radiological presentation, investigations, treatment, clinical and radiological outcome) and C (follow-up data).

The RITI registry includes a comprehensive set of questions organized into sections, illustrated below and in Figure 1: family and patient's history (section A), data on the thrombotic episode (section B), and follow-up data (section C).

A total of 2,668 questions are included in the RITI registry, although only a subset of these is shown for each specific patient, according to the age of onset of thrombosis and the thrombosis type, as appropriate (i.e., different subsets of questions in different thrombosis types and according to neonatal/pediatric age), especially as regards section B. Moreover, additional questions become available subject to the answer given to the main questions, and are otherwise not visible. Dropdown answers are used in most cases, and free text answers only in a minority.

### A. Demographics, family and personal history

This section comprises standardized questions for all patients, regardless of the type of thrombotic event and the age at onset.

#### Demographics

Date of birth, sex and race.

#### Family history

Thrombotic events and thrombophilia in other family members.

#### Personal history

Gestational age at birth, type of delivery, psycho-motor development, growth. Past medical issues and treatments.

### B. Thrombotic event

This is the core section of the registry, where the most important and detailed data are collected. Due to the diversity of features between different types of thrombosis events (neonatal/pediatric, cerebral/systemic, arterial/venous), this section contains thrombosis type-specific questions that are not applicable or relevant in other types of thrombosis. Below we provide a general overview of the data collected.

#### General data

Type of thrombosis (cerebral or systemic, arterial or venous), age at thrombosis event, date of diagnosis, signs and symptoms of thrombosis, hospital admission, intensive care unit admission. If the thrombotic event occurred in neonatal age, an additional section is made visible, collecting more detailed perinatal data: maternal age, previous pregnancies and fetal losses (if any), spontaneous or medically assisted pregnancy, maternal pregnancy and peri-partum infections, other pregnancy conditions, vaginal swab, premature rupture of membranes, intrapartum antibiotic prophylaxis, single or twin pregnancy, placental disorders, amniocentesis and chorionic villus sampling.

#### Risk factors

Arteriopathy, cardiac disease, infections, kidney or liver disease, neurocutaneous syndromes, autoimmune disorders, tumors, hematological disorders, thrombophilia, metabolic disorders, iatrogenic factors (medications, catheters, others), cardiac catheterization, interventional radiology, surgery (including general, cardiac, neurosurgery or others), organ transplant, extracorporeal membrane oxygenation, dialysis, other risk factors. If the thrombotic event occurred in neonatal age: need for resuscitation, hypoxic-ischemic encephalopathy, organ malformations and others. For catheter-related thrombosis: date of catheter insertion and removal, time between catheter insertion and thrombosis diagnosis, and between thrombosis diagnosis and catheter removal, arterial or venous catheterization, specific site of catheterization, catheter apex position, reason for catheterization, catheter type, caliber and material, number of lumens, insertion type, anticoagulant prophylaxis, catheter-related infections.

#### Clinical presentation

For cerebral thrombosis (AIS, CSVT): symptoms at presentation, pediatric National Institute of Health Stroke Scale (pedNIHSS) in the acute phase, history of transient ischemic attacks (TIA). For systemic thrombosis (arterial and venous): symptomatic or asymptomatic presentation, type of symptoms, thrombosis site, vessel involved.

#### Bloods

Complete blood count, electrolytes, pH, liver and kidney function tests, inflammatory markers, coagulation markers, microbiology searches, thrombophilia screening.

#### Radiology

For cerebral thrombosis (AIS, CSVT): type of parenchymal neuroimaging (CT/MRI/transfontanellar ultrasound) and its result, type of brain and neck vascular imaging (CT/MRI) and its result (arterial/venous territory, specific vessel involved), arteriography, transcranial and neck vessel doppler sonography, parenchymal and vascular localization of the infarct, presence of hemorrhagic lesions. For systemic thrombosis (arterial and venous): doppler ultrasound, CT, MRI, angiography, echocardiography, electrocardiogram, chest x-ray, abdominal ultrasound, other.

#### Other investigations

Echocardiography (trans-thoracic, trans-esophageal, bubble test), electrocardiogram, electroencephalogram.

#### Treatment

Fibrinolysis [tissue plasminogen activator (t-PA), urokinase (UK)], anticoagulants (warfarin, acenocoumarol, rivaroxaban, dabigatran, apixaban, fondaparinux, edoxaban, unfractioned heparin, low molecular weight heparin, other), antiaggregants (acetilsalycilic acid, ticlopidine, clopidogrel, other), other treatments (protein C, antithrombin, lepirubine, pentasaccaride, bivalirudine, argatroban, fresh frozen plasma, caval filter, embolectomy/thrombectomy, surgical revascularization, angioplasty, central venous catheter removal, peripheral catheter removal, other), treatment adverse reactions (thrombocytopenia, bleeding, other).

#### Outcome at discharge

Admission duration, occurrence of thrombosis relapse before discharge, neurological outcome at discharge in case of cerebral thrombosis [death, neurological deficits, pediatric stroke outcome measure (PSOM)], clinical sequelae in case of systemic thrombosis, radiological outcome.

### C. Follow-up

#### Clinical and radiological data

Date and age at follow-up, thrombosis relapse, quality of life, neurological clinical outcome, neurological sequelae and PSOM score in case of cerebral thrombosis, systemic clinical outcome and sequelae in case of systemic thrombosis, radiological follow-up, ongoing treatments.

This section allows for periodical update by the enrolling physician (i.e., follow-up at 3-month, 6-month, etc.).

## Overview of current RITI contributors and data, RITI literature and other registry-related activities

### RITI contributors

**Figure 2:**
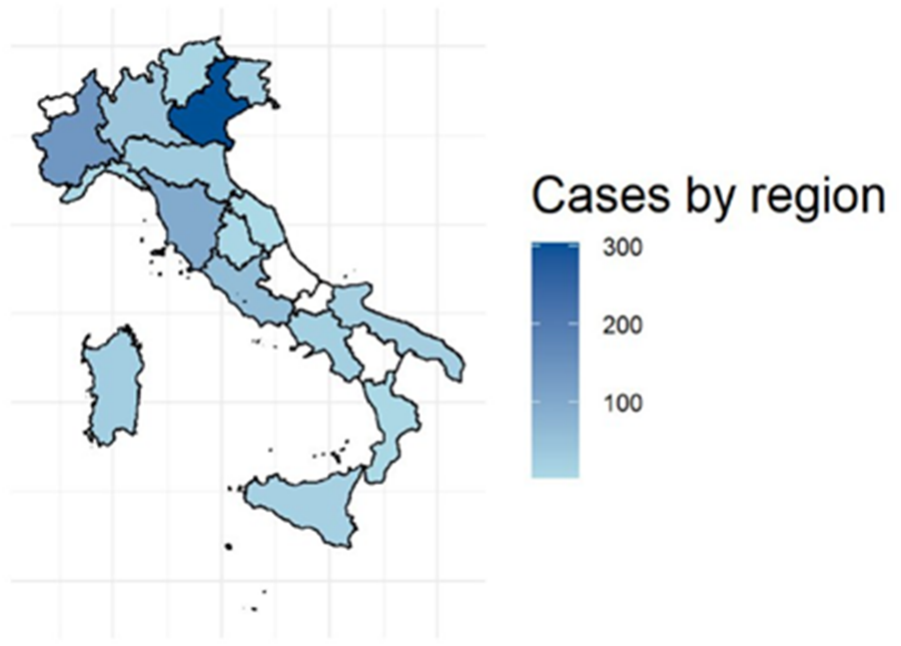
Map of Italy, showing the number of enrolled cases for each region. Shades of blue reflect the activity of a specific region. Regions in white are not actively contributing to the RITI registry activity at the present time.

At present, 48 Italian primary, secondary and tertiary pediatric and intensive care units have joined the RITI with 85 medical doctors from 16 out of 20 Italian regions being actively involved in data collection (Figure 2), and a total of 160 medical doctors registered in the RITI (see Appendix). These latter medical doctors are mostly afferent to pediatric units (69%), followed by neonatal units (21%), and other departments (10%); subspecialties include pediatrics (43%), child neuropsychiatry (25%), neonatology (21%), internal medicine (5%), neurology (3%), hematology (2%), emergency medicine (1%).

### RITI data

**Figure 3:**
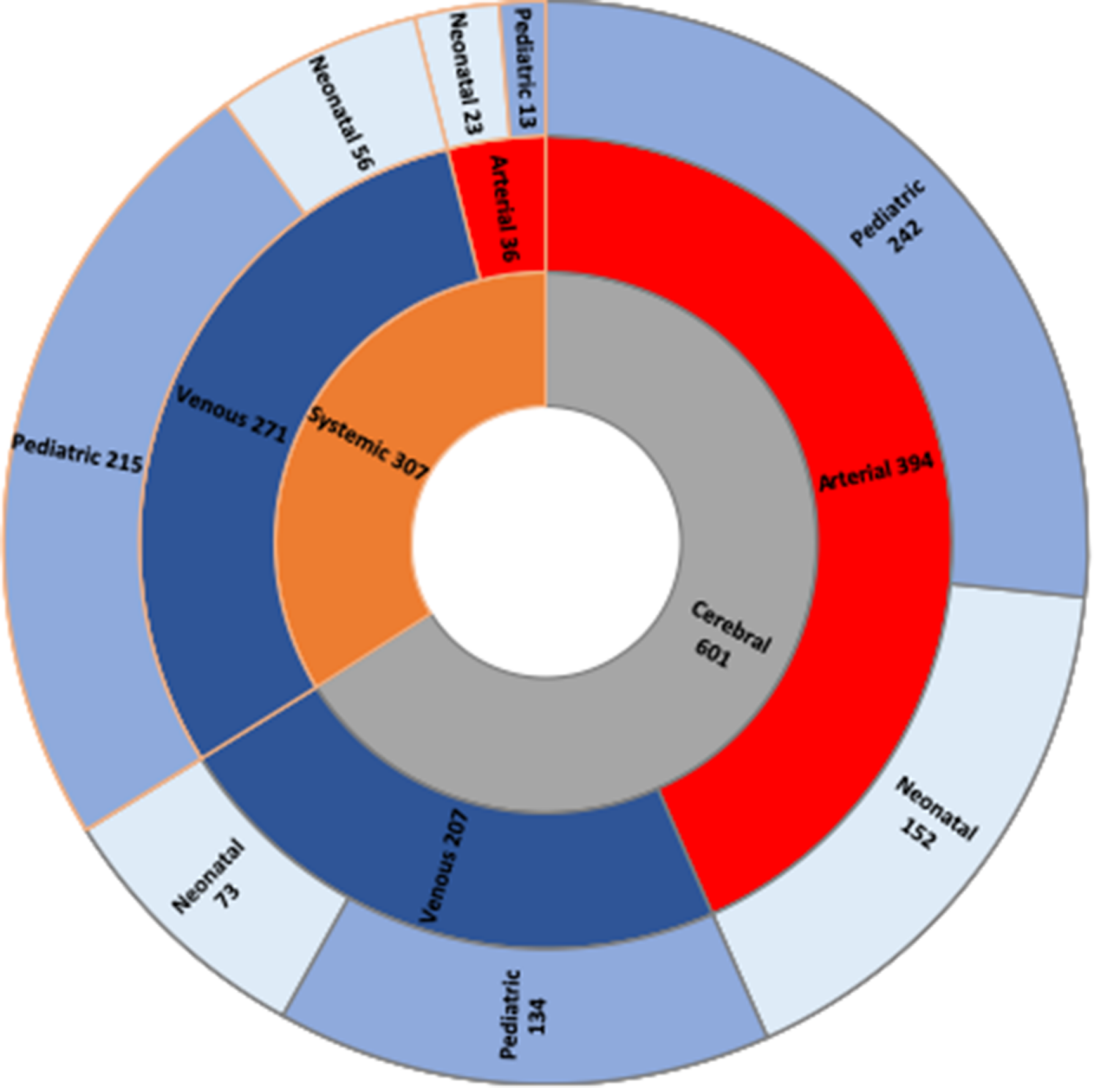
Thrombotic events stratified in three layers. The innermost layer shows the stratification between cerebral thrombotic (gray) and systemic (orange) events can be seen. The middle layer shows the distribution of arterial (red) and venous (blue) events. Finally, in the outermost layer the age stratification is displayed: neonatal events (light blue) and paediatric events (purple).

Data presented below are subject to heterogenous availability, therefore denominators may vary. Since 2007, 1,001 neonatal and pediatric patients affected by thrombosis have been retrospectively and prospectively identified (thrombotic events occurred between 1996 and 2022): 579/1,001 (57.8%) were male and the remaining 422/1,001 (42.2%) were female; age at first thrombotic event was median 0.9 years (interquartile range 0.02–6.6 years) (data on age at thrombotic event available in 960/1,001 patients). Data on age at onset and thrombosis type were simultaneously available in 909/1,001 patients: 225/909 (24.8%) had neonatal cerebral thrombosis, 79/909 (8.7%) had neonatal systemic thrombosis, 377/909 (41.5%) had pediatric cerebral thrombosis and 228/909 (25.1%) had pediatric systemic thrombosis. Data on treatment were available in 910/1,001 of the total population: 647/910 of these received antithrombotic treatment (71.1%). Follow-up data were available in 480/1,001 patients: in these, length of follow-up was median 16 months (interquartile range 6–37.8 months) (≥12 months in 290/480). Outcome data were available in 840/1,001 patients, and death was reported in 20/840 (2.4%) of these. Thrombosis recurrence was reported in 61/1,001 patients (6.1%). Stratification of thrombotic events is displayed in Figure 3.

### RITI literature

Publications on RITI data so far have covered the areas of pediatric cerebral thrombosis (25), neonatal systemic thrombosis (36), pediatric venous thromboembolism (37), and pediatric systemic catheter-related venous thromboembolism (24). The main data derived from these publications are provided in Table 4.

**Table 4:** Overview of RITI publications.

First author and publication year (reference)	Suppiej 2015 (25)	Saracco 2016 (36)	Giordano 2018 (37)	Lasagni 2022 (24)
Cerebral/Systemic thrombosis	Cerebral	Systemic	Systemic	Systemic
Arterial/Venous/Other	Arterial (AIS) + Venous (CSVT)	Arterial + Venous + Intracardiac (ICT)	Venous (DVT, PE)	Venous (CVC-VTE)
Age (neonatal or pediatric)	Pediatric	Neonatal	Pediatric	Pediatric
Nr of patients included (total nr of thrombotic events)	155 (170)	75 (75)	88 (92)	76 (78)
Main findings	• Most common modes of presentation in AIS: hemiparesis, seizures and speech disturbances. • Most common modes of presentation in CSVT: headache, seizures and lethargy. • Most common etiologies is AIS: underlying chronic diseases, vasculopathy and cardiopathy. • Most common etiologies is CSVT: underlying chronic diseases and infection. • Time to diagnosis: >24 h in 46% AIS and 59% CSVT (longer time to diagnosis compared to other registries).	• 41/75 events (55%) were VTE, 22/75 (29%) AT, and 12/75 (16%) ICT. • 65% patients were male. • 71% patients were preterm. • Thromboembolism diagnosis on the first day of life in 19/75 (25%): in this “early onset” group, prenatal-associated risk conditions (maternal/placental disease) were reported in 70% and inherited thrombophilia in 33%. • Postnatal risk factors in 73%: infections and central vascular catheters in 56% and 54% VTE, respectively, and in 67% ICT vs. 27% AT (<.05). • Thromboembolism-related death: 4%. • Thromboembolism-related sequelae: 16%.	• 85 DVTs and 7 PE. • Prevalence peak: age 1–5 years and age 10–18 years. • A central venous line was the main risk factor (55% of VTEs); surgery (not cardiac) (25%), concomitant infections (23%) and malignancy (22%) were the clinical conditions most often associated with VTE onset. • Diagnostic delay >24 h in 37% of VTEs. • Doppler ultrasound was the most widely used test for the objective diagnosis of DVT (87%). • Antithrombotic therapy: 96% of VTEs, mainly low molecular weight heparin (60%). • Recurrences: 2%. • Thrombotic syndrome: 8.5%.	• 67 non-cardiac VTEs (86%) and 11 ICTs (14%); median age at onset 19 and 17 months, respectively. • Percutaneously placed CVCs in 85% (56/66) and surgically in 15% (10/66). • PICCs were used in 47% (31/66), partially implanted catheters in 42% (28/66), non-implantable catheters in 7% (5/66), and totally implanted catheters (Port) in 2% (1/66). • Symptomatic CVC-VTEs in 77% (60/78), incidental finding in 23%. • Median time between CVC insertion and symptom onset: 10 days in non-cardiac VTEs and 39 days in ICTEs. • The veins of the lower extremities were affected in 52% (43/73). • Anti-thrombotic treatment: 96% (75/78). • Recurrence: 2.6% (2/76). • At discharge, post-thrombotic syndrome in 13.5% (5/37), CVC replacement in 10.8% (4/47), and ischemic necrosis with toe finger amputation in 2.7% (1/37). • 3 patients died due to an underlying condition; no CVC-VTE-related deaths.

AIS, arterial ischemic stroke; AT, arterial thromboembolism; CSVT, cerebral sinovenous thrombosis; CVC-VTE, catheter-related VTE; DVT, deep venous thromboses; ICT, intracardiac thromboembolism; PE, pulmonary embolism; PICC, peripherally inserted central catheter; VTE, venous thromboembolism.

### Other RITI activities

The RITI working group is also involved in an educational activity with the final scope of improving the knowledge on neonatal and pediatric thrombosis in Italy, and supporting enrolment of patients in the registry.

In particular, the RITI steering group organizes monthly online educational meetings, open to all RITI collaborators, to ensure constant update on thrombotic diseases, discuss clinical cases, and to monitor newly enrolled cases in the RITI. Members of the RITI working group throughout Italy are encouraged to take an active role in these meetings, by taking turns in giving the presentations.

## Critical appraisal of the RITI registry: a reflection on its journey, challenges and pitfalls

Creation, maintenance, improvement and promotion of the RITI registry have been a collective effort, journey and learning experience for the whole multidisciplinary team involved. Here, we want to reflect on our experience so far and share it, with focus on pitfalls, limitations and main challenges. These are listed in the six subsections below, each followed by an additional subsection suggesting potential strategies (“Useful interventions”) to overcome them.

### Registry designing

As previously mentioned, a radical change in the registry structure initially created in 2007 was undertaken in 2017, to transition from enrolment of thrombotic *events* to enrolment of *patients* with thrombotic events. This was felt strongly necessary in order to allow for a more accurate collection of follow-up data and to conform to the main international and national registries (Table 3). The change involved an enormous effort from the whole team, due to the necessity of creating a new informatic platform and manual data reinsertion in the new platform, therefore it represented a tremendous halt in the progression of patient enrolment and in the scientific production. Although, it was also felt as an opportunity for improvement, and the chance was taken to carry out a thorough critical revision of the registry structure and questions in order to update it, improve it and ameliorate its user-friendliness. With new literature evidence becoming available, modified disease classifications put forward and adopted, new diagnostic techniques and treatments used, it is in the possible natural history of certain registry features to become obsolete. While a periodic registry update to accommodate for these changes may be somewhat necessary and relatively feasible for prospectively enrolled patients, this is hardly possible for patients already enrolled.

#### Useful interventions

To minimize the need for changing the registry structure and questions, in the registry designing and planning phase the potential for future modifications or additions should be encompassed, for example aiming for an adequate balance between drop-down questions (easier to analyze) and free-text fields (allowing for inclusion of details not contemplated in the original set of questions). Moreover, post-insertion re-grouping or classifications may be possible at the time of data analysis, if originally inserted data are thorough and detailed enough to allow for this. Finally, it comes without saying that registry designing should be preceded by an accurate literature review and supported by adequate knowledge of the field, as quality data collection is strongly dependent on quality and appropriateness of questions.

### Registry diffusion

Among the main current challenges related to the RITI registry is promoting patient enrolment among a wider pool of Italian physicians, with the aim for the RITI to be representative of the whole Italian territory rather than the expression of a few larger centers only. Indeed, while the current number of involved physicians and Italian centers and regions is considerable, in several cases only a few patients have been enrolled, less than expected for the single centers, even after taking into account the likely different incidence of thrombotic events between smaller and larger centers, and the centralization of more severe patients to centers with higher expertise. Multiple factors likely contribute to this, including the possible heterogeneous vocation for research among different centers, and most importantly the lack of resources dedicated to research and the time-consuming nature of data insertion in the registry platform. Regarding this latter point, it should be noticed that the RITI platform is characterized by a high level of detail, making it a valid source of accurate and precise information but also making patient enrolment a lengthy process (i.e., about 40–60 min per patient), which cannot easily be afforded in resource-constrained environments.

#### Useful interventions

A national registry such as the RITI is a powerful tool shared by all physicians willing to contribute to it, and should be felt as such. Registry-related initiatives are key to promote registry diffusion and participation; the involvement of regional or local scientific societies could also help serve this purpose, reaching a more capillary diffusion. Similarly, educational events aiming at improving clinicians’ awareness on the condition of interest (i.e., pediatric thrombosis) would have indirect favorable repercussions on registry participation.

If registry resources allow it, another effective strategy to favor registry dissemination and participation include the availability of registry-trained personnel to travel to participating centers and offer training and support with patient enrolment and data insertion (8, 17).

Beside patient enrolment, an active role in data analysis, research and scientific production should be encouraged and made possible to a large base of active physicians participating to the registry, rather than centralized. This is indeed a very crucial step, but one of the most rewarding, and may have dramatic positive repercussions on the registry vitality and growth.

### Incomplete data

Despite the large amount of data collected in the RITI, only an extremely limited set of information is mandatory for the enrolment of a specific patient, in order to maximize patient inclusion even in the cases when not all data are available. This inevitably results in incomplete data availability for different subsets of information. Data may be missing due to permanent unavailability (i.e., information not collected or not retrievable at the time of the clinical interview, or in case a certain investigation was not carried out, or was performed at a different center and the result could not be retrieved), or temporary unavailability (i.e., outstanding test results at the time of patient enrolment, or follow-up information).

A subset of RITI data particularly prone to incompleteness is that of follow-up. Indeed, enrolment of a patient in the RITI ideally requires a multi-step commitment by the enrolling physician extending beyond the acute phase, that is to update the follow-up data over time. This is a critical step to provide long-term follow-up information on outcome and thrombosis recurrence, which is often lacking in the literature. Although, this also represents a time-consuming activity often going neglected and is a strong limitation in the RITI.

#### Useful interventions

Periodic automatic reminders to enrolling physicians represent a simple strategy to favor data completeness and insertion of follow-up information. Although, data quality control (see below) and personalized, *ad hoc* communications are far more likely to reach their goals.

### Inconsistent or inaccurate data

Data inconsistency between different data fields is also possible, due to errors filling in the registry fields, or potentially inaccurate diagnosis.

#### Useful interventions

Due to possible data inconsistency or incompleteness (see also subsection 3) above), centralized data verification is an important step in improving the quality of a registry data. While permanently unavailable data are unlikely to be retrieved, an accurate process of data control and verification may have potential for improving completeness of temporary unavailable data, and consistency and accuracy of information. In our experience, this has proved a very demanding task, that should be carried out on a patient-by-patient basis, by an expert in the field (and ideally, by an expert team), and should involve contacting the enrolling physicians to chase missing data and clarify the identified issues with them. At a retrospective glance, this has been mostly done as a preparatory work for data analysis on specific subsets of populations in the RITI; although, a regular periodic process of quality control would be ideal.

### Registry expenses and funding

Creation and maintenance of a registry are expensive, mostly due to the costs of the informatic platform and website. As regards the RITI registry, funds derived from non-profit organization dedicated to thrombosis (ALT, Associazione per la Lotta alla Trombosi e alle malattie cerebrovascolari: https://www.trombosi.org/, and GIRTI, Gruppo Italiano per il Registro della Trombosi Infantile, https://www.girti.it/) and research resources made it possible, beside the voluntary work of several clinical and non-clinical researchers.

#### Useful interventions

When planning and designing a registry, current and future expenses related to informatic support, data analysis, scientific dissemination and other should be carefully evaluated and taken into account. Funding may be derived for example from research resources (i.e., in academic settings), grants, patients’ organizations and funds allocated to non-profit organizations. Given the multicenter nature of registries, costs may potentially be shared by multiple centers.

### Main limitations

Due to the heterogenous patient enrolment on the Italian territory and the voluntary nature of participation to the registry, differently to other models such as the Swiss Neuro-Paediatric Stroke Registry (27) (Table 3), data derived from the RITI registry cannot be used for epidemiological information. Besides, hospital discharge codes do not seem to be completely adequate to retrieve patients with thrombosis in our experience (although this may not apply to other countries).

Moreover, RITI data is subject to selection bias: many of the centers involved are tertiary care hospitals caring for very specific subsets of patients, such as children with congenital heart disease who can be more easily prone to develop incident and relapsing thrombotic events. Furthermore, most of the patients included in the RITI are children who experienced cerebral thrombosis, due to the relatively high participation of pediatric neurologists to the registry.

Therefore, the retrospective observational nature of the registry and the limitations described above, among others, should be kept in mind when handling data derived from the registry, and its results should be interpreted with caution.

#### Useful interventions

Epidemiological significance of the registry could be reached in case patient enrolment is made mandatory, such as the Swiss Neuro-Paediatric Stroke Registry (27), or if a capillary registry participation is obtained. Nonetheless, registries based on voluntary participation can still provide valuable non-epidemiological data by pooling together a larger number of patients with rare conditions than would be possible in individual centers. Registry data also allow the comparison of different epochs, for example as regards outcomes before and after a certain treatment has become available or recommended, or different areas/regions, and they can light a spark for future and more focused studies with different designs.

### Conclusions and future directions

Overall, and despite these limitations and challenges, the RITI registry represents a model for multicenter collaboration on a nation-wide scale and the study of rare disorders. The registry may also serve as a tool to support the growth of individual centers through a collaborative discussion on case management, pursuing the final aim of improving the clinical care and outcome of neonatal and pediatric patients with thrombosis through the creation of a network of dedicated professionals and increasing knowledge and awareness on this disorder.

The RITI is currently one of the largest national registries of pediatric thrombosis in Europe, and includes the multidisciplinary expertise of its contributors among its main strengths. Aiming for a growing participation both in patient enrolment and data analysis, increasing scientific production and reaching greater information completeness and quality and are among the main short-term RITI goals. Potential future implementations, such as those to reflect evolving literature definitions and evidence, and to include original patient data for central review, such as radiology imaging, would be welcome future ambitious goals of the registry.

## Data Availability

The raw data supporting the conclusions of this article will be made available by the authors, without undue reservation.

## Appendix

*Collaborators of the Italian Registry of Infantile Thrombosis (RITI)*

– Accorsi Patrizia
– Aceto Gabriella
– Agnoletti Gabriella
– Agostini Manuela
– Alfarano Angela
– Altieri Elena
– Amador Carolina
– Antonelli Camilla
– Arena Vittoria
– Asta Francesca
– Baggio Laura
– Ballardini Elisa
– Baracetti Margherita
– Baraldi Eugenio
– Barberis Laura
– Barisone Elena
– Basso Anne Letizia
– Battajon Nadia
– Bersani Iliana
– Biddeci Giada
– Biffanti Roberta
– Bonardi Claudia Maria
– Bonaudo Roberto
– Boniver Clementina
– Boscarol Gianluca
– Bottino Roberto
– Bravar Giulia
– Brizzi Ilaria
– Brolatti Noemi
– Braguglia Annabella
– Guaragni Brunetta
– Bugin Samuela
– Calvo Pier Luigi
– Capasso Antonella
– Capodiferro Donatella
– Cappelleri Alessia
– Cascarano Maria Teresa
– Casellato Susanna
– Casini Tommaso
– Catarzi Serena
– Cavaliere Elena
– Cavicchiolo Maria Elena
– Celestino Silvia
– Celle Maria Elena
– Centonze Nicola
– Cerutti Alessia
– Chakrokh Roksana
– Offer Chiara
– Chiodin Elisabetta
– Chirico Gaetano
– Chukhlantseva Natalia
– Cifarelli Paola
– Cinelli Giulia
– Coinu Marisa
– Colonna Clara
– Comito Donatella
– Corato Alessandra
– Cordelli Duccio Maria
– Crichiutti Giovanni
– Cursio Ida
– Dagri Arianna
– De Maria Beatrice
– Del Borrello Giovanni
– Di Rienzo Francesca
– Doglioni Nicoletta
– Dolcemascolo Valentina
– Dotta Andrea
– Drigo Paola
– Drimaco Pietro
– Ellero Serena
– Falcone Alessandra
– Fantauzzi Ambra
– Farinasso Daniela
– Ferilli Michela
– Festa Silvia
– Fischer Maximilian
– Foiadelli Thomas
– Fotzi Ilaria
– Francavilla Rosa
– Freschi Paola
– Gaffuri Marcella
– Gallo Elena
– Gamalero Lisa
– Gandioli Claudia
– Garuccio Sergio
– Gentile Diletta
– Ghionzoli Marco
– Giliberti Paola
– Greco Filippo
– Guariento Chiara
– Guidotti Isotta
– Iodice Alessandro
– Janes Augusta
– Laghi Elena
– Lampugnani Elisabetta
– Lassandro Giuseppe
– Laverda Anna Maria
– Lazzerotti Alessandra
– Lo Tartaro Meragliotta Patrizia
– Lombardini Martina
– Lorenzon Eleonora
– Mainini Nicoletta
– Massoud Michela
– Materia Valeria
– Mattera Raffaele
– Mauro Isabella
– Melani Federico
– Meli Mariaclaudia
– Messina Giovanni
– Monticone Sonia
– Moras Marzia
– Negro Ilaria
– Olzai Giorgio
– Pancani Simone
– Pandolfi Maria
– Passariello Annalisa
– Passarini Alice
– Passone Eva
– Pastorino Myriam
– Pegoraro Veronica
– Pennoni Serena
– Perilongo Giorgio
– Pozzessere Anna
– Pruna Dario
– Pusiol Anna
– Putti Maria Caterina
– Rabbone Ivana
– Radicioni Maurizio
– Renna Salvatore
– Ricci Maria Luisa
– Rimini Alessandro
– Rivellini Sara
– Rustioni Gianluca
– Salvadori Sabrina
– Santoiemma Valentina
– Santoro Nicola
– Schiavulli Michele
– Sebellin Sofia
– Sesta Michela
– Soffiati Massimo
– Sorbo Monica
– Spanedda Giuseppina
– Stangalini Valeria
– Stasolla Salvatore
– Tanzi Giorgia
– Testa Tiziana
– Teutonico Federica
– Timpani Giuseppina
– Toldo Irene
– Trapani Sandra
– Vaccari Roberto
– Vecchi Marilena
– Vento Giovanni
– Veraldi Daniele
– Villa Giovanna
– Visintin Gianluca
– Zambelloni Cesare
– Zellini Francesco

*Main Italian Centers involved in the Italian Registry of Infantile Thrombosis (RITI)*

– ASST Spedali Civili of Brescia, Brescia
– Azienda Ospedaliera Bianchi-Melacrino-Morelli di Reggio Calabria, Reggio Calabria
– Azienda Ospedaliera Policlinico Di Bari, Bari
– Bambin Gesù Children’s Hospital, Roma
– Central Teaching Hospital of Bolzano, Bolzano
– Giannina Gaslini Hospital, Genova
– Niguarda Hospital, Milano
– Ospedale Civile SS. Annunziata, Sassari
– San Bortolo Hospital, Vicenza
– San Matteo Hospital, Pavia
– Santa Chiara Hospital, Trento
– ULSS 2 Marca Trevigiana, Treviso
– ULSS 8 Berica, Vicenza
– University Hospital “Azienda Ospedaliero-Universitaria Meyer”, Firenze
– University Hospital Città della Salute e della Scienza, Torino
– University Hospital of Modena, Modena
– University Hospital of Padova, Padova
– University Hospital of Udine, Udine
– University Hospital of Verona, Verona
– University of Bari, Bari
– University of Bologna, Bologna
– University of Cagliari, Cagliari
– University of Catania, Catania
– University of Ferrara, Ferrara
– University of Napoli “Federico II”, Napoli
– University of Perugia, Perugia
