# Corrigendum: The new Italian registry of infantile thrombosis (RITI): a reflection on its journey, challenges and pitfalls

**DOI:** 10.3389/fped.2024.1372754

**Published:** 2024-01-31

**Authors:** Maria Federica Pelizza, Matteo Martinato, Anna Rosati, Margherita Nosadini, Paola Saracco, Paola Giordano, Matteo Luciani, Laura Ilardi, Donatella Lasagni, Angelo Claudio Molinari, Rossana Bagna, Antonella Palmieri, Luca Antonio Ramenghi, Massimo Grassi, Mariella Magarotto, Federica Magnetti, Andrea Francavilla, Giuseppe Indolfi, Agnese Suppiej, Chiara Gentilomo, Roberta Restelli, Antonella Tufano, Daniela Tormene, Jacopo Norberto Pin, Clarissa Tona, Davide Meneghesso, Lidia Rota, Marta Conti, Giovanna Russo, Giulia Lorenzoni, Dario Gregori, Stefano Sartori, Paolo Simioni, Collaborators of the R.I.T.I. (Italian and Registry of Infantile Thrombosis), Accorsi Patrizia

**Affiliations:** ^1^ Paediatric Neurology and Neurophysiology Unit, Department of Women’s and Children’s Health, University Hospital of Padova, Padova, Italy; ^2^ Master in Pediatrics and Pediatric Subspecialties, University of Padova, Padova, Italy; ^3^ Unit of Biostatistics, Epidemiology and Public Health, Department of Cardiac-Thoracic-Vascular Sciences and Public Health, University of Padova, Padova, Italy; ^4^ Department of Statistics, Computer Science, Applications “G. Parenti”, University of Firenze, Firenze, Italy; ^5^ Neuroscience Center of Excellence, Children’s Hospital Anna Meyer, University of Firenze, Firenze, Italy; ^6^ Paediatric Haematology Unit, Department of Paediatrics, University Hospital “Città Della Salute e Della Scienza”, Torino, Italy; ^7^ Department of Biomedical Science and Human Oncology, Aldo Moro University of Bari-Giovanni XXIII Hospital, Bari, Italy; ^8^ Department of Paediatric Hemato-Oncology and Cell and Gene Therapy, Bambino Gesù Children’s Hospital, Roma, Italy; ^9^ Neonatal Intensive Care Unit, Niguarda Ca’ Granda Hospital, Milano, Italy; ^10^ Paediatric Unit, Children’s Hospital Anna Meyer, University of Firenze, Firenze, Italy; ^11^ Regional Reference Center for Hemorrhagic Diseases, IRCCS Giannina Gaslini Children’s Hospital, Genova, Italy; ^12^ Neonatal Intensive Care Unit, University Hospital “Città Della Salute e Della Scienza”, Torino, Italy; ^13^ Department of Paediatric Emergency, IRCCS Giannina Gaslini Children’s Hospital, Genova, Italy; ^14^ Neonatal Intensive Care Unit, IRCCS Giannina Gaslini Children’s Hospital, Genova, Italy; ^15^ Neonatal Intensive Care Unit, Department of Women’s and Children’s Health, University Hospital of Padova, Padova, Italy; ^16^ NEUROFARBA Department, Children’s Hospital Anna Meyer, University of Firenze, Firenze, Italy; ^17^ Section of Pediatrics, Department of Medical Sciences, University of Ferrara, Ferrara, Italy; ^18^ Paediatric Unit, “Dell'Angelo” Hospital, Venezia, Italy; ^19^ Regional Reference Centre for Coagulation Disorders, Department of Clinical Medicine and Surgery, Federico II University Hospital, Napoli, Italy; ^20^ General Internal Medicine and Thrombotic and Haemorrhagic Unit, University Hospital of Padova, Padova, Italy; ^21^ Paediatric Nephrology, Dialysis and Transplant Unit, Department of Women’s and Children’s Health, University Hospital of Padova, Padova, Italy; ^22^ Cardiovascular Prevention Centre, Humanitas Research Hospital, Milano, Italy; ^23^ Child Neurology Unit, Department of Neuroscience, Bambino Gesù Children’s Hospital, Rome, Italy; ^24^ Unit of Pediatric Onco-Haematology, Department of Clinical and Experimental Medicine, University of Catania, Catania, Italy

**Keywords:** thrombosis, stroke, children, pediatric, registry, thromboembolism, neonatal

A Corrigendum on The new Italian registry of infantile thrombosis (RITI): a reflection on its journey, challenges and pitfalls Pelizza MF, Martinato M, Rosati A, Nosadini M, Saracco P, Giordano P, Luciani M, Ilardi L, Lasagni D, Molinari AC, Bagna R, Palmieri A, Ramenghi LA, Grassi M, Magarotto M, Magnetti F, Francavilla A, Indolfi G, Suppiej A, Gentilomo C, Restelli R, Tufano A, Tormene D, Pin JN, Tona C, Meneghesso D, Rota L, Conti M, Russo G, Lorenzoni G, Gregori D, Sartori S, Simioni P and Collaborators of the R.I.T.I. (Italian Registry of Infantile Thrombosis) (2023). Front. Pediatr. 11:1094246. doi: 10.3389/fped.2023.1094246

**Error in the Appendix**

In the published article, there was an error in the appendix. An author name was incorrectly written as Foidaelli Thomas. The correct spelling is Foiadelli Thomas. The correct appendix appears below.

**Appendix**

*Collaborators of the Italian Registry of Infantile Thrombosis (RITI)*

– Accorsi Patrizia

– Aceto Gabriella

– Agnoletti Gabriella

– Agostini Manuela

– Alfarano Angela

– Altieri Elena

– Amador Carolina

– Antonelli Camilla

– Arena Vittoria

– Asta Francesca

– Baggio Laura

– Ballardini Elisa

– Baracetti Margherita

– Baraldi Eugenio

– Barberis Laura

– Barisone Elena

– Basso Anne Letizia

– Battajon Nadia

– Bersani Iliana

– Biddeci Giada

– Biffanti Roberta

– Bonardi Claudia Maria

– Bonaudo Roberto

– Boniver Clementina

– Boscarol Gianluca

– Bottino Roberto

– Bravar Giulia

– Brizzi Ilaria

– Brolatti Noemi

– Braguglia Annabella

– Guaragni Brunetta

– Bugin Samuela

– Calvo Pier Luigi

– Capasso Antonella

– Capodiferro Donatella

– Cappelleri Alessia

– Cascarano Maria Teresa

– Casellato Susanna

– Casini Tommaso

– Catarzi Serena

– Cavaliere Elena

– Cavicchiolo Maria Elena

– Celestino Silvia

– Celle Maria Elena

– Centonze Nicola

– Cerutti Alessia

– Chakrokh Roksana

– Offer Chiara

– Chiodin Elisabetta

– Chirico Gaetano

– Chukhlantseva Natalia

– Cifarelli Paola

– Cinelli Giulia

– Coinu Marisa

– Colonna Clara

– Comito Donatella

– Corato Alessandra

– Cordelli Duccio Maria

– Crichiutti Giovanni

– Cursio Ida

– Dagri Arianna

– De Maria Beatrice

– Del Borrello Giovanni

– Di Rienzo Francesca

– Doglioni Nicoletta

– Dolcemascolo Valentina

– Dotta Andrea

– Drigo Paola

– Drimaco Pietro

– Ellero Serena

– Falcone Alessandra

– Fantauzzi Ambra

– Farinasso Daniela

– Ferilli Michela

– Festa Silvia

– Fischer Maximilian

– Foiadelli Thomas

– Fotzi Ilaria

– Francavilla Rosa

– Freschi Paola

– Gaffuri Marcella

– Gallo Elena

– Gamalero Lisa

– Gandioli Claudia

– Garuccio Sergio

– Gentile Diletta

– Ghionzoli Marco

– Giliberti Paola

– Greco Filippo

– Guariento Chiara

– Guidotti Isotta

– Iodice Alessandro

– Janes Augusta

– Laghi Elena

– Lampugnani Elisabetta

– Lassandro Giuseppe

– Laverda Anna Maria

– Lazzerotti Alessandra

– Lo Tartaro Meragliotta Patrizia

– Lombardini Martina

– Lorenzon Eleonora

– Mainini Nicoletta

– Massoud Michela

– Materia Valeria

– Mattera Raffaele

– Mauro Isabella

– Melani Federico

– Meli Mariaclaudia

– Messina Giovanni

– Monticone Sonia

– Moras Marzia

– Negro Ilaria

– Olzai Giorgio

– Pancani Simone

– Pandolfi Maria

– Passariello Annalisa

– Passarini Alice

– Passone Eva

– Pastorino Myriam

– Pegoraro Veronica

– Pennoni Serena

– Perilongo Giorgio

– Pozzessere Anna

– Pruna Dario

– Pusiol Anna

– Putti Maria Caterina

– Rabbone Ivana

– Radicioni Maurizio

– Renna Salvatore

– Ricci Maria Luisa

– Rimini Alessandro

– Rivellini Sara

– Rustioni Gianluca

– Salvadori Sabrina

– Santoiemma Valentina

– Santoro Nicola

– Schiavulli Michele

– Sebellin Sofia

– Sesta Michela

– Soffiati Massimo

– Sorbo Monica

– Spanedda Giuseppina

– Stangalini Valeria

– Stasolla Salvatore

– Tanzi Giorgia

– Testa Tiziana

– Teutonico Federica

– Timpani Giuseppina

– Toldo Irene

– Trapani Sandra

– Vaccari Roberto

– Vecchi Marilena

– Vento Giovanni

– Veraldi Daniele

– Villa Giovanna

– Visintin Gianluca

– Zambelloni Cesare

– Zellini Francesco

*Main Italian Centers involved in the Italian Registry of Infantile*

*Thrombosis (RITI)*

– ASST Spedali Civili of Brescia, Brescia

– Azienda Ospedaliera Bianchi-Melacrino-Morelli di Reggio

Calabria, Reggio Calabria

– Azienda Ospedaliera Policlinico Di Bari, Bari

– Bambin Gesù Children’s Hospital, Roma

– Central Teaching Hospital of Bolzano, Bolzano

– Giannina Gaslini Hospital, Genova

– Niguarda Hospital, Milano

– Ospedale Civile SS. Annunziata, Sassari

– San Bortolo Hospital, Vicenza

– San Matteo Hospital, Pavia

– Santa Chiara Hospital, Trento

– ULSS 2 Marca Trevigiana, Treviso

– ULSS 8 Berica, Vicenza

– University Hospital “Azienda Ospedaliero-Universitaria

Meyer”, Firenze

– University Hospital Città della Salute e della Scienza, Torino

– University Hospital of Modena, Modena

– University Hospital of Padova, Padova

– University Hospital of Udine, Udine

– University Hospital of Verona, Verona

– University of Bari, Bari

– University of Bologna, Bologna

– University of Cagliari, Cagliari

– University of Catania, Catania

– University of Ferrara, Ferrara

– University of Napoli “Federico II”, Napoli

– University of Perugia, Perugia

The authors apologize for this error and state that this does not change the scientific conclusions of the article in any way. The original article has been updated.

